# Urinary NGAL Ratio Is Not a Sensitive Biomarker for Monitoring Acute Tubular Injury in Kidney Transplant Patients: NGAL and ATI in Renal Transplant Patients

**DOI:** 10.1155/2012/563404

**Published:** 2012-12-27

**Authors:** Jessica K. Kaufeld, Wilfried Gwinner, Irina Scheffner, Hermann G. Haller, Mario Schiffer

**Affiliations:** Division of Nephrology, Hannover Medical School, Carl Neuberg Straße 1, 30625 Hannover, Germany

## Abstract

Urinary neutrophil gelatinase-associated lipocalin (uNGAL) is known to predict the prolonged delayed graft function after kidney transplantation. We examined the relation of uNGAL with histological findings of acute tubular injury (ATI). Analyses were made in biopsies taken at 6 weeks, 3 months, and 6 months after kidney transplantation. uNGAL was measured in the spot urines, normalized to urinary creatinine excretion, and correlated to biopsy findings and clinical, laboratory, and demographic variables. Controls included healthy individuals, individuals after kidney donation and ICU patients with acute kidney failure. Renal transplant recipients without ATI did not display elevated uNGAL levels compared to the healthy controls. Transplant patients with ATI had a higher uNGAL excretion at 6 weeks than patients without ATI (27,435 versus 13,605 ng/g; *P* = 0.031). This increase in uNGAL was minor compared to ICU patients with acute renal failure (2.05 × 106 ng/g). Patients with repeated findings of ATI or severe ATI did not have higher urinary NGAL levels compared to those with only one ATI finding or moderate ATI. Female recipient gender and urinary tract infection were identified as potential confounders. uNGAL has a relation with histological signs of acute tubular injury. The usability of this biomarker in renal allograft recipients is limited because of the low sensitivity.

## 1. Introduction

Acute kidney injury early after transplantation may arise from the donor's condition, prolonged cold ischemia time, and early posttransplant injuries like rejection and drug toxicity. Immediate clinical correlates of this injury may be delayed graft function or a less-than-optimal glomerular filtration rate. However, acute kidney injury carries adverse long-term consequences as patients with acute tubular injury early posttransplantation have an inferior long-term allograft function [1]. Therefore, markers, which facilitate early diagnosis of acute kidney injury or even provide prognostic information, would be helpful in the posttransplant care of these patients. 

Traditionally, serum parameters as creatinine and urine output are used to monitor kidney function in transplanted patients. However, a rise in serum creatinine already implies a significant amount of kidney damage, which limits its ability to detect impaired graft function at early stages. Recently, novel biomarkers such as Kim-1, IL-18, and neutrophil gelatinase-associated lipocalin (NGAL) have been proven useful in detecting nontransplant acute kidney damage [2]. NGAL, a 25-kDa protein, is a member of the lipocalin family, which is expressed in many human tissues, including the kidney. In children and adults with cardiopulmonary surgery, NGAL was specifically and early elevated in those patients that developed acute renal failure a few days after surgery [3]. Parikh et al. reported that urinary NGAL could be an early predictor of DGF after kidney transplantation [4]. In a previous study by Mishra et al. with immunochemical staining of renal allografts, a correlation was established between an increased expression of NGAL and prolonged cold ischemia time, elevated serum creatinine levels, and dialysis requirement [5]. In another study, urinary NGAL was more accurate in predicting dialysis within one week of transplantation than serum creatinine [6]. 

Acute kidney injury primarily affects renal tubules and NGAL is produced in injured tubular epithelial cells. Therefore, we addressed the question whether urinary NGAL correlates with histologically confirmed acute tubular injury using protocol biopsies taken within the first six months after transplantation. 

## 2. Materials and Methods

### 2.1. Study Population and Sample Collection

The study population consisted of 140 adult kidney transplant recipients randomly chosen from our protocol biopsy archive (*n* = 35 patients who never had any signs of ATI and *n* = 105 patients with at least one finding of ATI during the protocol biopsy program). Controls included 9 healthy individuals (2-kidney controls), 13 individuals after kidney donation (1-kidney control), and 5 ICU patients with acute kidney failure. Renal protocol biopsies are regularly performed 6 weeks and 3 and 6 months after kidney transplantation in the transplant center of the Medical School of Hannover since 2001. All patients are eligible for the protocol biopsies unless certain medical conditions like bleeding disorders and other comorbidities advice against a biopsy. In addition to these regular protocol biopsies, biopsies for cause are performed in case of unclear allograft impairment. Only data of patients undergoing regular protocol biopsies were included in this study. Demographic and clinical data of patients participating in this program were collected prior to and at the time of transplantation. The demographics for patients with and without ATI are depicted in Table 1. For patients without ATI *n* = 23 samples were taken at 6 weeks, *n* = 17 at 3 months, and *n* = 16 at 6 months after transplantation. For patients with signs of ATI *n* = 44 samples were taken at 6 weeks, *n* = 3 samples at 3 months, and *n* = 55 samples at 6 months. After transplantation, clinical data and routine laboratory results were collected corresponding to the time points of the three protocol biopsies. Spot urine was frozen in −80°C at time of the biopsy. Biopsies were evaluated according to the updated Banff classification [7]. Data and sample collections were performed with an informed consent of the patients and with the approval of the ethics board of the Medical School Hannover. 

### 2.2. NGAL Detection

Urinary NGAL was measured in the spot urine using the Quantikine human lipocalin-2/NGAL Immunoassay (Cat.no. DLCN 20 by R&D Systems), according to the manufacturer's protocol. Urinary NGAL excretion was normalized to urinary creatinine excretion to correct for differences in NGAL due to urine dilution. Urinary NGAL excretion is presented as the amount of urinary NGAL in ng per g of urine creatinine.

### 2.3. Statistical Analyses

Descriptive data were reported as medians with ranges. Comparisons of categorical data between groups were performed with Fisher's exact test and the chi-square test for two or more samples. Numerical data were compared with the Kruskal-Wallis test and the Mann-Whitney test. Linear regression analyses were performed with log transformed NGAL values as the dependent variable and the demographical, clinical, and laboratory factors that had been significantly different in univariate analyses. Variables from the univariate analyses were separately examined for the samples at 6 weeks and 6 months using the backward selection. SPSS statistical software package version 18.0 (SPSS Inc., Chicago, IL) was used for statistical analysis. Statistical significance was assumed for *P* < 0.05. Note that for better visualisation the logarithm to base 10 was used in all figures with NGAL results, because these values were highly variable and not normally distributed.

## 3. Results

### 3.1. uNGAL Excretion in Patients after Kidney Transplantation Remains Constant over Time and Is Comparable to Healthy Controls

**Figure 1:**
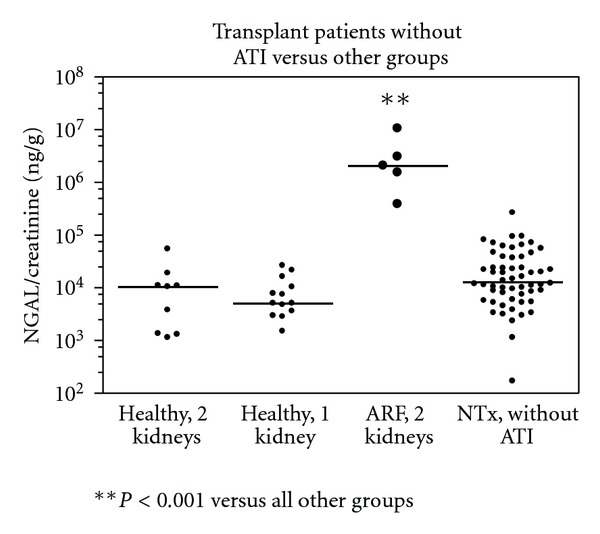
Urinary NGAL/creatinine ratios in transplanted patients versus other groups. Shown are the urinary NGAL/creatinine levels of the two control groups (healthy individuals with 2 (*n* = 9) or 1 kidney (*n* = 13)) versus ICU patients with acute renal failure (ARF, 2 kidney (*n* = 5)) and transplanted patients without ATI (*n* = 56). Patients with acute renal failure had significantly higher urinary NGAL levels compared to the other groups (*P* < 0.001).

**Figure 2:**
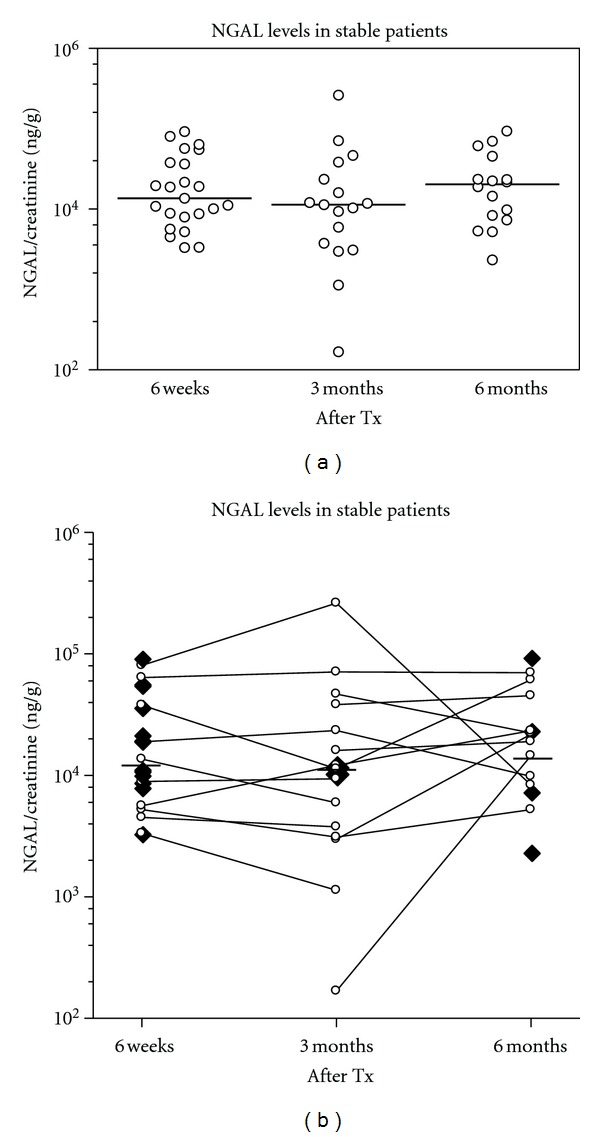
(a) NGAL excretion in stable patients without ATI at 6 weeks, 3 months, and 6 months after transplantation. Shown is the urinary NGAL/creatinine ratio at 6 weeks (*n* = 23), 3 months (*n* = 17), and 6 months (*n* = 16) in patients after kidney transplantation. (b) Individual course of NGAL excretion of the stable patients at 6 weeks, 3 months, and 6 months after transplantation. Depicted are single measurements (*n* = 13 at 6 weeks, *n* = 2 at 3 months, *n* = 4 at 6 months) as single black squares and serial measurements (6 weeks to 3 months: *n* = 4 patients, 3 months to 6 months: *n* = 5 patients, and 6 weeks to 3 months and 6 months: *n* = 6 patients) as open circles connected by lines. Total measurements at 6 weeks: *n* = 23, at 3 months: *n* = 17, and 6 months: *n* = 16.

First we compared the uNGAL excretion levels of renal allograft recipients without ATI to different control groups. There was no significant difference in uNGAL excretion in transplanted patients versus healthy individuals with 1 kidney (i.e., kidney donors) and healthy individuals with 2 kidneys (Figure 1). In nontransplanted patients with acute renal failure, the NGAL/creatinine ratio was significantly higher 2.0490e + 006 ng/g (382498 to 1.0470e + 007) compared to healthy individuals with one or two kidneys and to transplanted patients without signs of ATI in the biopsies up to 6 months after transplantation (*P* < 0.001). Subsequent analyses represent a cross-sectional study of samples where not all patients provided urine samples at every time point, showing no time-associated differences in samples taken at 6 weeks, 3 months, and 6 months after transplantation (Figure 2(a)).  Figure 2(b) depicts the dynamic change of uNGAL excretion at 6 weeks, 3 months and 6 months after transplantation in individual patients without ATI.

### 3.2. uNGAL Is Not Sensitive to Detect ATI in Transplanted Patients

**Figure 3:**
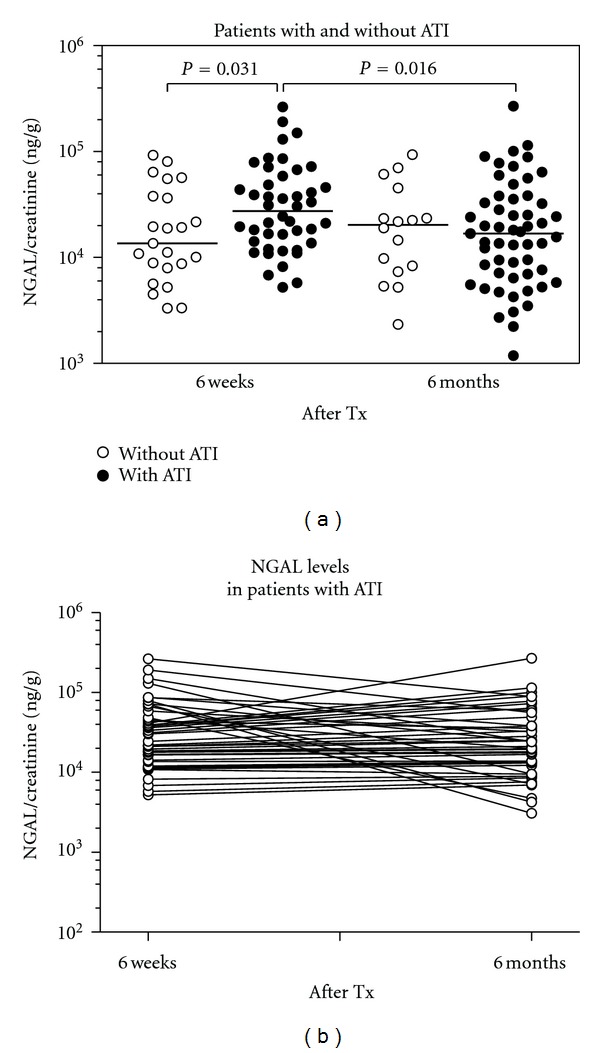
(a) Comparison of urinary NGAL/creatinine ratios in patients with and without ATI after kidney transplantation. Patients with ATI 6 weeks (*n* = 44) after transplantation have significantly higher urinary NGAL levels than patients without ATI (*n* = 23). In patients with ATI, the urinary NGAL excretion at 6 months (*n* = 55) is significantly lower than at 6 weeks. No difference in uNGAL ratios was seen between patients with and without ATI at 6 months. (b) Individual course of NGAL levels in patients with ATI at 6 weeks and 6 months after transplantation. Patients with singular measurement are presented by single values only (dots; *n* = 11 patients for the 6-month measurements). Serial measurements: *n* = 44 patients for 6 weeks and 6 months (connected by lines).

**Figure 4:**
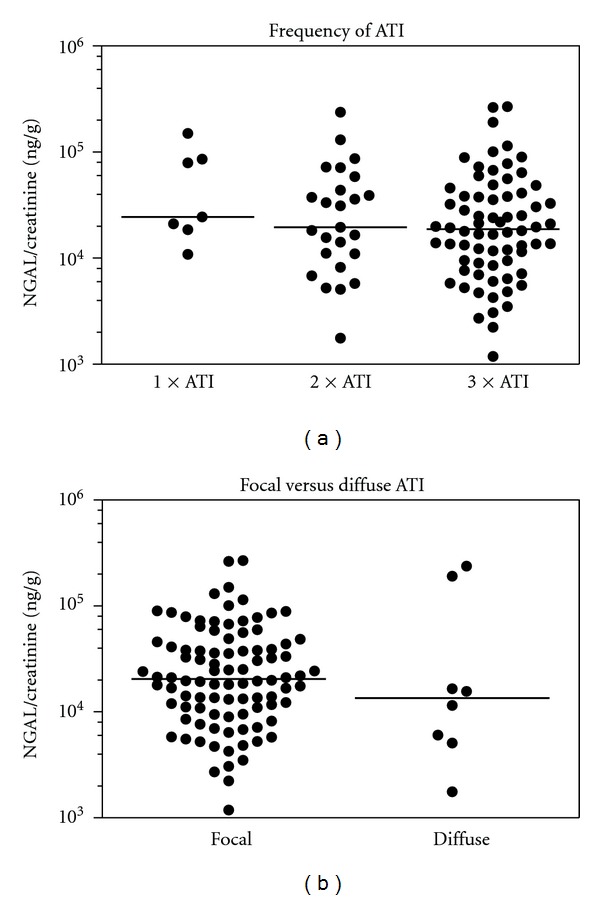
(a) Urinary NGAL/creatinine ratio depending on the frequency of ATI. Shown is the urinary NGAL/creatinine ratio depending on whether patients had 1 time (*n* = 7), 2 times (*n* = 25), or 3 times (*n* = 70) findings of ATI in biopsies taken while being followed in the biopsy program. (b) Urinary NGAL/creatinine ratio in Tx patients with focal versus diffuse ATI. No significant differences were found in the urinary NGAL excretion of patients with focal (*n* = 94) versus diffuse (*n* = 8) ATI findings after Tx.

Next, we tested the sensitivity of urinary NGAL as a biomarker to indicate ATI in transplanted patients. At six weeks, patients with ATI had urinary NGAL values that were two-fold higher than the values from patients without ATI in the biopsies, yet there was a considerable overlap of values between the two groups. At 6 months, no differences between patients with and without ATI were apparent (Figure 3(a)).  Figure 3(b) depicts the dynamic change of uNGAL excretion in patients with ATI. Multiple findings of ATI did not correlate with higher uNGAL levels (Figure 4(a)). Moreover, the extent of ATI, whether diffuse or focal, did not cause a difference of the NGAL ratios (Figure 4(b)). 

### 3.3. Association of NGAL Levels with Clinical Variables and Exploration of Potential Confounders

In an explorative analysis, we correlated NGAL levels to allograft damage (e.g., acute tubular injury, delayed graft function, donor age) and to additional clinical, laboratory, and demographic variables (for a complete list see Supplementary Materials available online at doi:10.1155/2012/563404). The main findings of the univariate analysis are depicted in Table 2. Female recipient gender was associated with significant higher NGAL ratios at 6 weeks and 6 months after transplantation. Regarding donor gender, a numerical trend towards higher NGAL values for allografts from female donors became apparent at 6 months after transplantation. A lower GFR at the time of sampling was weakly associated with higher NGAL ratios at 6 months. Furthermore, we analyzed whether acute impairment of the allograft function at the time of biopsy had any relation to NGAL levels. Serum creatinine levels at the day of biopsy showed changes relative to the baseline values before the biopsy from −29 to 129% in patients without acute tubular injury and from −40 to 60% in patients with acute tubular injury. However, a correlation of the change in creatinine with uNGAL excretion could not be established for these patients (*r* ^2^ ≤ 0.1; data not shown).

Isometric vacuolization and prevalence of chronic graft changes (cGrade>0) according to the current BANFF classification at 6 weeks after transplantation were associated with significantly higher uNGAL levels. However, after 6 months, uNGAL ratios were lower in patients with isometric vacuolization. In addition, patients with urinary tract infection at the time of sampling had significantly higher uNGAL ratios at 6 weeks as well as 6 months (Table 2).

Next, linear regression analyses were performed with log transformed NGAL values to determine the contribution of the identified variables of the explorative univariate analysis to the observed NGAL levels. Significant variables from the univariate analyses were separately examined for the samples at 6 weeks and 6 months using the backward selection. For the 6-week samples (Table 3(a)), presence of urinary tract infection and isometric vacuolization of tubular epithelial cells in the concomitant biopsy were associated with higher NGAL levels (*r* ^2^ = 0.296; unadjusted R Square of model fit). Recipient gender was not a significant factor in the regression model at 6 weeks (*r* ^2^ = 0.138; *P* = 0.131). In the 6-month samples (Table 3(b)), urinary tract infection, female recipient gender, and absence of isometric vacuolization were associated with higher NGAL levels (*r* ^2^ = 0.357). 

Since the univariate analysis indicated a relation between donor and recipient gender and NGAL levels, the possible gender combinations are additionally presented (Table 4). Significant differences were observed between the male donor/male recipient group compared to all other possible combinations, except the female/male combination at six weeks (*P* = 0.20). 

## 4. Discussion

Acute tubular injury (ATI) is a frequent finding in biopsies of renal allograft recipients and is linked to an inferior long-term allograft function [1]. In this study, we tested whether urinary NGAL (uNGAL) may be applicable as a sensitive and noninvasive biomarker to monitor ATI in renal allograft recipients.

NGAL can be produced locally in the kidney by injured tubular cells and can be secreted by activated neutrophils/macrophages or inflamed vasculature [8]. In a recent experimental study, Paragas et al. demonstrate that uNGAL originates predominately from the kidney. This group generated an NGAL reporter mouse, which showed a close correlation between tubular stress and uNGAL [9]. Thus, uNGAL levels could reflect kidney damage in real time, as discussed by Mori and Nakao [10]. In animal models, NGAL has shown to be the most upregulated gene and overexpressed protein with a high predictive value for acute kidney injury [11–13]. 

We established the baseline uNGAL excretion in our transplanted patient cohort without findings of ATI. uNGAL levels in this group were comparable to healthy controls and remained stable 6 weeks, 3 months, and 6 months after transplantation. As proper controls for patients with a renal allograft, we collected sample material from healthy individuals with one kidney (donors with normal kidney function), in addition to healthy controls with 2 kidneys. Our observation of a tendency towards a higher NGAL excretion in healthy individuals with 2 kidneys versus 1 kidney may point to a confounding effect by the kidney mass. This finding should be confirmed with a larger cohort. 

In patients with ATI, median uNGAL ratios were significantly higher at 6 weeks after transplantation than in patients without signs of ATI. While these findings may indicate a correlation of uNGAL excretion to ATI in patients early after transplantation, we could not demonstrate increased uNGAL levels in patients with ATI at 6 months after transplantation. On the contrary, median uNGAL levels in patients with ATI were even lower at 6 months compared to the controls without ATI. Further analyses showed no differences of NGAL excretion depending on the frequency of ATI or the distinction between diffuse versus focal ATI. NGAL levels were weakly inversely correlated to the eGFR; however, no link could be established between uNGAL levels and acute rises in serum creatinine at the time of ATI diagnosis. Urinary NGAL levels have been shown to correlate with histopathological alterations in IgAN patients [14]. Tubulointerstitial lesions in these patients were characterized by tubular atrophy, interstitial fibrosis, and inflammatory cell infiltration. Acute tubular injury in our transplanted patients differed from these features as it was defined by epithelial swelling with lucency of the cytoplasm, loss of brush border, and/or luminal dilatation with flattening of the epithelium and cytoplasmatic vacuolization [1]. Furthermore, even though a 2-fold increase in NGAL was present in our patients at 6 weeks, the substantial overlap with values from patients without ATI precludes the use of NGAL as a sensitive marker for kidney injury in transplant patients. Hollmen et al. found that high urinary NGAL levels until day 14 after transplantation in patients with early graft function (EGF) were associated with worse kidney function at 3 weeks, but not at 3 months or 1 year after kidney transplantation [15]. Most studies validating NGAL as a marker for allograft function emphasize its predictive value first and foremost in early urine or serum samples, up to 2 weeks after transplantation [4, 5]. NGAL showed to be an early marker for AKI and subsequent dialysis or DGF in transplanted patients by means of immuneohistochemical staining, urinary excretion levels, and serum levels [5, 6, 15, 16]. In our patient population with ATI findings, a considerable proportion (68.8%) had delayed graft function and need for posttransplantation, yet this was not reflected by a relevantly elevated urinary NGAL value at 6 weeks posttransplantation. Further, in patients with stable graft function, subclinical tubulitis was associated with higher NGAL levels [17]. In our regression analyses, uNGAL ratios correlated significantly with isometric vacuolization 6 weeks after transplantation, which could be related to immunosuppressant toxicity and damage [18], whereas isometrical vacuolization at 6 months was associated with lower NGAL levels. This raises the question whether uNGAL ratios in transplanted patients really reflect the ongoing tubular damage or whether NGAL may be more of an indicator for regenerative capacities of the kidney. 

Plasma NGAL measurements can be influenced by numerous clinical variables, such as hypertension [19], systemic infections [20], age [21], and a higher validity in children. In our linear regression analyses, we confirmed that in patients with concomitant urinary tract infection at 6 weeks and at 6 months posttransplantation uNGAL ratios were significantly higher. This could be related to the leukocytes present in the urine as shown by Decavele et al. who demonstrated a correlation of one urinary leukocyte to an increase of 12 pg of uNGAL [22]. One other important but unexpected finding of our study was that female patients with grafts from female kidney donors as well as female graft recipients with male or female donors had higher uNGAL ratios compared to male recipients with kidneys from male donors. Further parameters such as coronary heart disease and blood transfusion before transplantation could be correlated to higher NGAL levels after 6 months but not at 6 weeks after transplantation. These variables showed no significant association to uNGAL levels in subsequent multivariate analyses but may indicate other potential confounders in transplanted patients.

The limitations to our study are that we present a single-centre study and that urine samples were stored at −80°C for more than a year and some protein degradation may have occurred during the freeze-thaw cycle thus, this study should be confirmed with immediate uNGAL measurements in fresh urine samples. In addition we do not have information about the uNGAL levels in our patients prior to transplantation or in the donors and some patients did not provide urine samples at all time points when protocol biopsies were taken. Thus, further studies are necessary to evaluate the diagnostic value of uNGAL measurements in patients after kidney transplantation.

In conclusion, even though NGAL may be useful in classifying patients with established AKI or glomerulonephritis in native kidneys or may be a predictor for delayed graft function and recovery early after kidney transplantation [14–16, 23], it is not a sensitive tool for monitoring for ATI at later times after transplantation. 

## Supplementary Material

Variables without statistical significance after correlation to urinary NGAL ratios in an explorative analysis.

## Figures and Tables

**Table 1:** Demographic and clinical data of patients with and without ATI.

	ATI	ATI+	All patients	*P* value
	*N* = 35	*N* = 105	*N* = 140	
Recipient				
Age (years)	49 ± 12 (49,20–69)	51 ± 12 (52,25–73)	51 ± 12 (50,20–73)	
Gender (% m/f)	65,7/34,3	61,9/38,1	62,9/37,1	
Retransplanted pat. (%)	8,6	10,5	10,0	
Preformed antibodies neg. (%)	94,3	93,3	93,5	
Lowest s-creatinine within the first 6 weeks of tx	160 ± 83	182 ± 87	176 ± 86	
Hypercholesteroemia before tx (%)	31,4	37,1	35,7	
Arterial hypertension before tx (%)	100	96,2	97,1	
Donor				
Age (years)	47 ± 15 (50,7−70)	51 ± 13 (52,16–77)	50 ± 14 (51,7–77)	
Gender (% m/f)	60/40	53,3/46,7	55/45	
Type				
Deceased (%)	82,9	95,2	92,1	0.029
Living (%)	17,1	4,8	7,9	
CMV IgG positive (%)	60	53,8	55,4	
s-creatinine (*μ*mol/L)	95 ± 36	93 ± 44	93 ± 42	
Graft factors at tx				
Cold ischemia time (h)	14 ± 7	17 ± 7	17 ± 7	0.032
Mean number of HLA mismatches	2,26 ± 1,6	2,4 ± 1,72	2,36 ± 1,68	
Initial function of the graft (%)	68,6	28,6	38,6	0.000
Need of dialysis following tx (%)	31,4	71,4	62,1	0.000
Immunosuppressive regimen (%)				
CyA-MMF-Steroids-IL2	37,1	31	32,6	
CyA-MMF-Steroids-ATG	0	1	0,7	
CyA-MMF-Steroids-no induction	0	3	2,2	
CyA-MMF-IL2	14,3	31	26,7	
CyA-MMF-ATG	0	1	0,7	
Other, IL2	28,6	14	17,8	
Other, ATG	11,4	7	8,1	
Other, no induction	8,6	12	11,1	

ATI−: patients without ATI; ATI+: patients with ATI; tx: transplantation; median and range of values in brackets; CyA: cyclosporine A; MMF: mycophenolate mofetil; ATG: antithymocyte globulin; IL2: interleukin 2 receptor antagonist.

**Table 2:** Urinary NGAL levels (NGAL/creatinine; (ng/g)), and associations with various clinical, laboratory and demographic variables. Results are separately reported for the 6-week and 6-month samples. The remaining variables which were tested without significant differences are reported in the Supplementary Materials.

	6 weeks		*P* value	6 months		*P* value
	Male	Female		Male	Female	
Donor gender	18,875 (*n* = 47)	23,910 (*n* = 37)	0.31	12,264 (*n* = 35)	21,785 (*n* = 37)	0.071
Recipient gender	18,242 (*n* = 54)	37,855 (*n* = 30)	0.011	11,491 (*n* = 44)	32,613 (*n* = 28)	0.000
GFR^a^	*R* = −0.204		0.066	*R* = −0.321		0.006

	6 weeks		*P* value	6 months		*P* value
	Not present	Present		Not present	Present	

Delayed graft function	18,875 (*n* = 13)	21,315 (*n* = 71)	0.218	17,554 (*n* = 45)	19,354 (*n* = 27)	0.866
Isometric vacuolization^b^	19,187 (*n* = 61)	31,224 (*n* = 23)	0.031	22,436 (*n* = 47)	9,480 (*n* = 25)	0.008
cGrade >0^b^	19,031 (*n* = 70)	39,575 (*n* = 14)	0.033	14,218 (*n* = 44)	21,115 (*n* = 28)	0.583
Urinary tract infection at the time of sampling	17,901 (*n* = 63)	50,548 (*n* = 14)	0.000	13,760 (*n* = 56)	64,889 (*n* = 6)	0.014
Panel reactive antibodies at transplantation > 0%	22,952 (*n* = 76)	11,696 (*n* = 7)	0.019	17,194 (*n* = 66)	33,929 (*n* = 6)	0.342
Blood transfusion before transplantation	19,530 (*n* = 57)	25,876 (*n* = 18)	0.449	13,286 (*n* = 47)	30,322 (*n* = 18)	0.022
MMF in the initial immunosuppression	27,842 (*n* = 31)	19,187 (*n* = 53)	0.245	24,208 (*n* = 32)	17,875 (*n* = 40)	0.019
Coronary heart disease before transplantation	16,502 (*n* = 70)	21,822 (*n* = 14)	0.116	19,354 (*n* = 61)	8,969 (*n* = 11)	0.043

^a^GFR calculated according to the Cockroft and Gault formula at the time of sampling, ^b^according to the BANFF classification, at the time of sampling.

**Table:** (a) Samples from 6 weeks.

Model component	Regression coefficient	SE	*P* value	Tolerance
Constant	5.029	0.224	<0.001	
Urinary tract infection	0.519	0.110	<0.001	0.980
Isometric vacuolization	0.272	0.091	0.004	1.0

**Table:** (b) Samples from 6 months.

Model component	Regression coefficient	SE	*P* value	Tolerance
Constant	4.364	0.338	<0.001	
Urinary tract infection	0.335	0.151	0.03	0.967
Female recipient	0.427	0.097	<0.001	0.970
Isometric vacuolization	−0.293	0.099	0.004	0.994

SE: standard error of the regression coefficient; tolerance indicates collinearity between the variables of mode I (1.0 = no collinearity). Calculated GFR's are based on the serum creatinine and Cockroft and Gault formula and square roots of GFR values are used for the analysis.

**Table 4:** Correlation of uNGAL ratios to the different donor/recipient gender combinations.

Gender combinations donor/recipient	NGAL in 6-week samples^a^ (ng/g)	NGAL in 6-month samples^b^ (ng/g)
Male/male	14,259	5,670
	*n* = 28	*n* = 20
Male/female	37,717^c^	32,893^e^
	*n* = 19	*n* = 15
Female/female	37,993^d^	24,050^e^
	*n* = 11	*n* = 13
Female/male	21,654	18,454^f^
	*n* = 26	*n* = 24

Urinary NGAL/creatinine ratios at 6 weeks and 6 months after transplantation according to the four combinations of donor and recipient gender. At both time points, differences between the four combinations were significant (^a^ *P* = 0.041 and ^b^ *P* < 0.001; the Kruskal Wallis test). Posttesting identified differences to the male donor/male recipient group (^c^ *P* = 0.035; ^d^ *P* = 0.017; ^e^ *P* < 0.001; ^f^ *P* = 0.002).
