# Supplementary material for: Urinary NGAL Ratio Is Not a Sensitive Biomarker for Monitoring Acute Tubular Injury in Kidney Transplant Patients: NGAL and ATI in Renal Transplant Patients
Source: J Transplant. 2012 Dec 27;2012:563404. doi: 10.1155/2012/563404 (PMC3543801; doi:10.1155/2012/563404)
Supplement: Supplementary file 1 — Variables without statistical significance after correlation to urinary NGAL ratios in an explorative analysis. [file 563404.f1.doc]

**Supplementary Materials: Variables that were correlated to NGAL in an explorative Analysis**

| **Basic data** | **Allograft-related factors a the time of sampling** |
| --- | --- |
| Recipient gender | Serum creatinine |
| Recipient age at transplantation | Volume of the graft |
| Time on dialysis prior to transplantation | Resistance index |
| Cause of endstage renal failure | Renal graft artery stenosis |
| Donor’s gender | Hydronephrosis of the allograft |
| Donor’s age |  |
| Donor type (deceased, living) | **Concomitant findings in the biopsy** |
| Additional pancreas transplantation | Acute tubular injury |
| Donor’s serum creatinine | Isometric vacuolization of tubular epithelia |
| Cold ischemia time | Tubulointerstitial calcification |
| Delayed graft function | Interstitial fibrosis and tubular atrophy |
|  | Polyoma nephropathy |
| **Immunological status** |  |
| HLA-mismatches at locus A, B and DR | **Medications** (at the time of sampling) |
| Preformed antibodies at transplantation | Immunosuppressive maintenance therapy |
| Previous transplantations | Angiotensin converting enzyme inhibitors |
| Blood transfusions before transplantation | Angiotensin receptor blockers |
| Pregnancies before transplantation | Beta-blockers |
| Induction therapy ( IL-2R abs or ATG) | Calcium channel blockers |
| Initial immunosuppressive combination | Peripheral alpha/beta-Blockers |
|  | Centrally acting antihypertensives |
| **Pre- or co-existing morbidities** | Diuretics |
| Arterial hypertension | Direct vasodilators |
| Coronary artery disease | Aspirin |
| Heart failure | Ticlopidine |
| Peripheral artery disease | Clopidogrel |
| Diabetes mellitus | Heparin |
| Cigarette smoking | Insulin |
| Hypercholesterolemia | Alpha-glucosidase antagonists |
| Hyperparathyroidism | Sulfonylurea/glitazones/biguanides |
| CMV antigenemia | Allopurinol |
| Hepatitis B | Benzbromarone |
| Hepatitis C | Vitamin D analogs |
| Urinary tract infections | Bisphosphonates |
| Other bacterial infections | Cotrimoxazole prophylaxis |
|  | Other antibacterial therapies |
|  | Ganciclovir/valganciclovir |
